# Supplementary material for: Norovirus Seroprevalence among Adults in the United States: Analysis of NHANES Serum Specimens from 1999–2000 and 2003–2004
Source: Viruses. 2020 Feb 5;12(2):179. doi: 10.3390/v12020179 (PMC7077181; doi:10.3390/v12020179)
Supplement: Supplementary file 1 [file viruses-12-00179-s001.pdf]

Norovirus Seroprevalence in Adults in the United States: Analysis of NHANES Serum Specimens from 1999-2000 and 2003-2004

Amy E. Kirby [1,3]*, Yvonne Kienast [1], Wanzhe Zhu [1], Jerusha Barton [1], Emeli Anderson [1], Melissa Sizemore [1], Jan Vinje [2], and Christine L. Moe [1]

Supplemental Material

Figure S1. Overall seroprevelance and serologic evidence of recent infection stratified by antigen, age group, and NHANES study cycle. Overall seroprevalence defined as O.D. ≥ 1.5. Evidence of recent infection defined as O.D. ≥ 3.0. Closed circles, 1999-2000; open circles, 2003-2004.

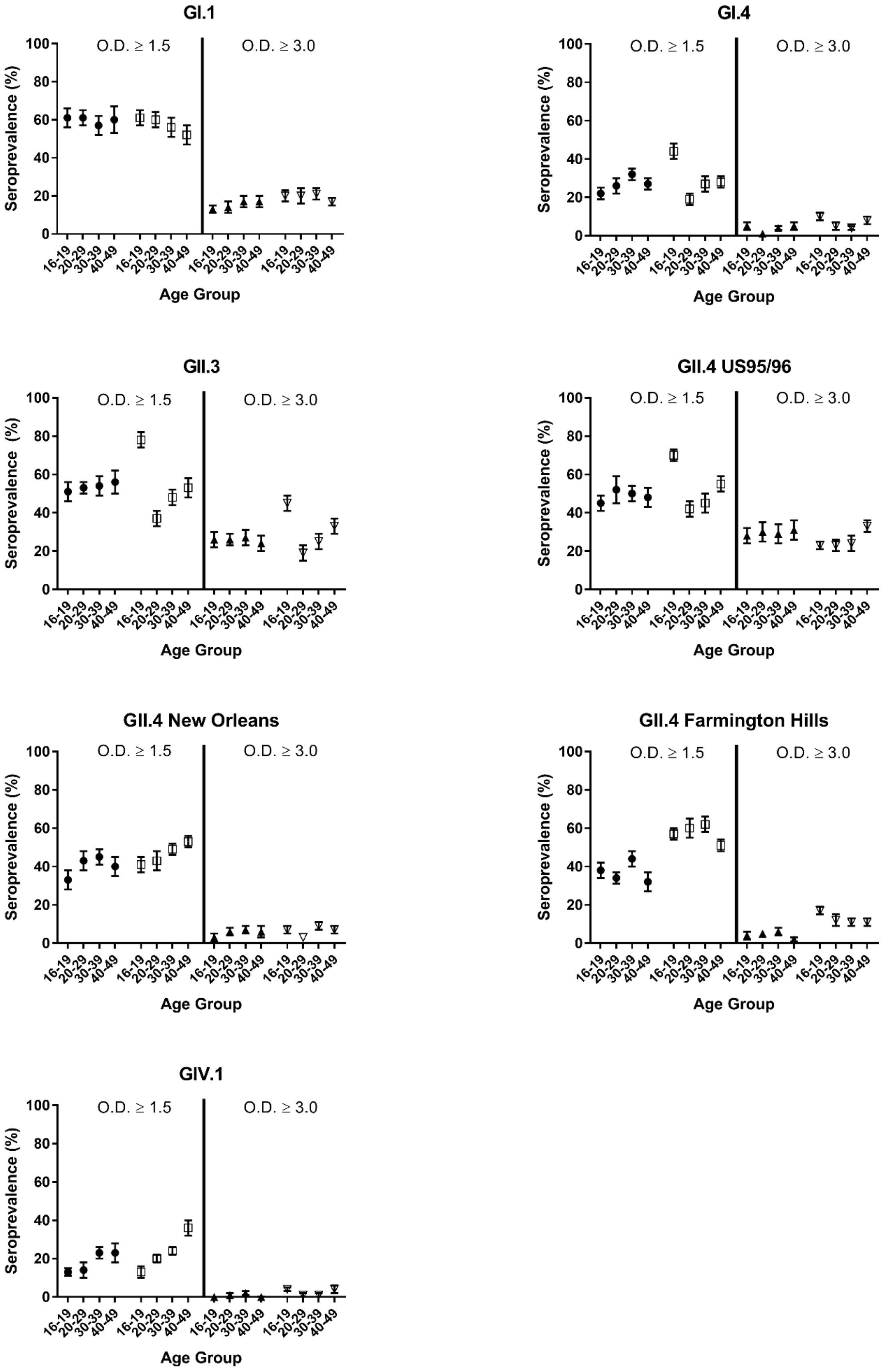

GI.1
GI.4
GII.3
GII.4 US95/96
GII.4 New Orleans
GII.4 Farmington Hills
GIV.1
O.D. ≥ 1.5
O.D. ≥ 3.0
Seroprevalence (%)
0
20
40
60
80
100
16-19
20-29
30-39
40-49
Age Group
